# Cancer-Specific Stress and Mood Disturbance: Implications for Symptom Perception, Quality of Life, and Immune Response in Women Shortly after Diagnosis of Breast Cancer

**DOI:** 10.5402/2012/608039

**Published:** 2012-12-20

**Authors:** Duck-Hee Kang, Na-Jin Park, Traci McArdle

**Affiliations:** ^1^University of Texas School of Nursing at Houston, 6901 Bertner Avenue, CNR No. 536, Houston, TX 77030, USA; ^2^University of Pittsburgh School of Nursing, 3500 Victoria Street, VB 415, Pittsburgh, PA 15261, USA; ^3^UAB Hospital, 184-A Russel Wing, 1813 6th Avenue South, Birmingham, AL 35249, USA

## Abstract

*Purpose*. To determine the levels of cancer-specific stress and mood disturbance in women shortly after diagnosis of breast cancer and to assess their associations with symptom perception, quality of life, and immune response. *Design*. Descriptive and correlational. *Sample and Setting*. One hundred women with newly diagnosed breast cancer were recruited from interdisciplinary breast clinics. *Methods*. Baseline data were collected using standardized questionnaires and established bioassay prior to the initiation of cancer adjuvant therapy. Blood samples were collected about the same time of day. *Results*. High cancer-specific stress was significantly correlated with high mood disturbance, which, in turn, was correlated with high symptom perception, poor quality of life, and an immune profile indicating high neutrophils and low lymphocytes. *Conclusions*. High cancer-specific stress and related mood disturbance show extensive negative relationships with multiple behavioral, clinical, and biological factors. *Implications for Nursing*. Routine screening for cancer-related stress and mood disturbance should be incorporated into nursing practice for all patients diagnosed with cancer. Given broad negative associations with other biobehavioral factors, early identification of patients at risk and provision and evaluation of stress and mood management programs may have a beneficial effect on subsequent health outcomes over time.

## 1. Introduction

Cancer diagnosis is a significant source of psychological stress/distress (hereinafter referred to as stress), followed by an extended period of stressful cancer treatment [1–3]. Not surprisingly, patients with a diagnosis of cancer report high psychological and emotional stress [4]. Although initial stress tends to decline over time for most patients, many others continue to suffer from high stress for years even after the successful completion of cancer treatment [1]. Studies indicate that up to 30% of patients report high stress levels years from breast cancer diagnosis and surgery [1, 5]. Some patients even experience posttraumatic stress disorder precipitated by cancer diagnosis [6, 7].

Mood disturbance, particularly of depression and anxiety, is common with cancer diagnosis and has been highly correlated with psychological stress [8, 9]. About 20–40% of cancer patients have reported significant levels of depressive mood and anxiety, and these patients typically have reported higher frequency and severity of clinical symptoms, including pain, fatigue, poor appetite, sleep disturbance, and poor quality of life [10–15]. In some studies, depression has been associated with higher cancer recurrence and cancer mortality, although this association has not been consistent in all studies [16–19].

Psychological stress may have multiple negative impacts on health outcomes. Chronic psychological stress has been linked to higher incidence of infections, accelerated aging, and greater cardiovascular diseases in diverse populations [20–22]. In patients with breast cancer, high psychological stress was associated with low physical and psychological quality of life [3, 23–25] and even a significantly shorter disease-free interval than women without major stress experience [26]. In addition, stress was found to decrease the efficacy of or resistance to chemotherapeutic agents in animal models [27, 28]. 

The psychoneuroimmunology literature clearly demonstrates that psychological and emotional stress induces significant alterations in various biological responses. The typical activation of the hypothalamic-pituitary-adrenal axis and the sympathetic nervous system may trigger a shift in immune cell traffics and facilitate inflammation via multiple neuroendocrine and immune pathways [25, 29–31]. Higher stress was correlated with poorer immune responses [32], and stress reduction seemed to improve immune responses (Natural killer cell activity) in women with breast cancer [33]. 

Neutrophils are the most numerous and constitute about 55–65% of the total leukocyte count, whereas lymphocytes constitute about 25–35% of the total leukocyte count in peripheral circulation. Neutrophils play an important role in the innate immunity and are the first line of defense mechanism against infectious agents or tissue injury. Neutrophils migrate into the inflamed site and ingest and destroy microorganisms through the formation of reactive oxygen species, hydrolytic enzymes, and antimicrobial polypeptides. Paradox of neutrophil biology, however, is that these same cells also participate in the pathology of various inflammatory conditions, such as rheumatoid arthritis, tumor initiation, and adult respiratory distress syndrome [34]. Lymphocytes control the acquired/adaptive immunity and play a major role in the regulation of cellular and humoral immunity. Although lymphocyte functions and its subset changes have been studied extensively, neutrophil responses have rarely been investigated in relation to psychosocial factors. In previous studies, academic stress was found to significantly increase neutrophil superoxide release, a proinflammatory substance [35, 36], supporting their role in inflammation under stress.

 Many stress-induced alterations in immune functions are thought to be mediated by changes in immune cell counts. The cell count changes may reflect leukocyte redistribution, not necessarily leukocyte destruction, most likely mediated by stress-related hormones [37]. Stress and glucocorticoid hormones, for example, tend to reduce the lymphocyte counts, whereas catecholamines tend to increase the neutrophil counts but decrease the lymphocyte counts in circulation [37–39]. Furthermore, investigators recently found that the neutrophil to lymphocyte ratio (NLR) had important implications for cancer outcomes. An elevated preoperative NLR, particularly a value greater than 4 or 5, was predictive of overall and cancer-specific survival and cancer recurrence in colorectal cancer patients [40, 41] and patients with malignant mesothelioma [42]. Similarly, in a large sample of patients with various types of cancer, an elevated NLR was an independent predictor for survival in all cancers studied [43].

The specific aims of this study, therefore, were to (1) assess the levels of cancer-specific stress and mood disturbance (including depression and anxiety) in women shortly after diagnosis of breast cancer and (2) examine the associations of cancer-specific stress and mood disturbance with symptom perception, quality of life, and immune cell counts (total leukocytes, neutrophils, lymphocytes, and NLR). Data were drawn from the parent study, in which the effects of an integrated intervention of cognitive behavioral stress management and exercise were examined in women with newly diagnosed breast cancer over a 1-year period. Only baseline data prior to the initiation of cancer adjuvant therapy and intervention were used for this present study. 

## 2. Materials and Methods

### 2.1. Design

This was a descriptive and correlational study based on the baseline data collected shortly after diagnosis of breast cancer in women prior to the start of cancer adjuvant therapy. 

### 2.2. Sample

Of more than 1,000 women screened, 213 women met the eligibility criteria, and 100 women enrolled in the parent study. Inclusion criteria for parent study were (1) new diagnosis of breast cancer, (2) plan to receive chemotherapy and/or radiotherapy, (3) age 30 years or older, (4) absence of known psychiatric illness, (5) no participation in other structured support or exercise programs, and (6) ability to comprehend and respond in English. Exclusion criteria were (1) pregnancy, (2) diffuse bony metastasis with high risk of pathologic fractures; and (3) no access to a telephone. The study protocol was approved by the institutional review board, and the study was conducted to maintain ethical standards. The sample size was based on power analysis of the parent study related to the most important functional immune parameter and intervention [44]. A total of 90 subjects were required to detect the group difference in the immune measure with 80% power and 5% alpha based on a 2-tailed *t*-test [45]. 

### 2.3. Setting and Data Collection

Participants were recruited by posting flyers in the interdisciplinary clinic areas, word of mouth, and invitations from clinic staff and research team. If patients showed initial interest in the study, research staff provided the details of the study including the purpose of the study, procedure and data collection plan, what were expected from them, and potential risks and benefits. When patients were ready to enroll, the written consent was obtained, and participants were stratified by cancer stage (stage I-IIB versus III and above) and randomized into either the intervention or the wait-list control group using a computer-generated randomization table. For all participants, the baseline data were collected before the beginning of cancer adjuvant therapy and intervention. Psychosocial and behavioral data were collected using the standardized and validated questionnaires, clinical information was obtained from medical records with prior permission, and immune cell counts were obtained from the laboratory services. All blood samples (15–20 mL) for immune responses were collected between 0830 and 1200 during their routine clinic visits by a phlebotomist in the clinic laboratory or by a trained nurse. 

### 2.4. Measurements

(1) Cancer-specific stress was measured with the Impact of Event Scale (IES, [46]). The IES is a 15-item validated standardized inventory to assess intrusive and avoidant thoughts and actions regarding cancer. Participants rated the intensity and frequency of occurrence in the past 7 days on a scale of 0 = not at all, 1 = rarely, 3 = sometimes, and 5 = often. The reliability was .85 in this study. 

(2) Mood disturbance was measured with a short version of the Profile of Mood States (POMS) [47]. The short version was designed for clinical use and included 37 items [48]. Participants rated each item on a 5-point Likert scale ranging from 0 = not at all to 5 = extremely. The inventory included six dimensions of mood disturbance: Tension-Anxiety, Depression-Dejection, Anger-Hostility, Fatigue-Inertia, Vigor-Activity, and Confusion-Bewilderment. Internal consistency reliability was reported to be .80–.91 for all dimensions. Total score was used in this study, and the reliability was .94.

(3) Symptom perception was measured with Memorial Symptom Assessment Scale [49]. The scale included 32 items in psychological, physical, and global dimensions. Participants rated each item on a rating scale ranging from 1 to 4 with a higher rating indicating more frequency, severity, and distress. Total score was used in this study, and the reliability was .82.

(4) Quality of life was measured with the Functional Assessment of Cancer Therapy Scale-B (FACT-B) [50]. The FACT-B is a 44-item questionnaire including physical, functional, social, emotional, relationship with doctor, and additional concerns specific to breast cancer. Participants rated each item on a rating scale ranging from 0 = not at all to 4 = very much. Total score was used in this study, and the reliability was .89.

(5) Demographic and clinical information was collected using a demographic information sheet and by extracting data from medical records with permission. 

(6) Immune cell counts were assessed with total leukocyte count with differentials serviced by the university affiliated hospital certified clinical laboratory.

### 2.5. Data Analysis

 Data were analyzed using the statistical package of PASW statistics v. 18 (SPSS Inc.). Descriptive data were examined to determine baseline levels of study variables. Correlations were determined with Pearson's correlation coefficients, and alpha level was set at .05. 

## 3. Results

### 3.1. Characteristics of Participants

The mean age of the participants was 48.8 years (Table 1). Participants were relatively well educated with 70% of them having some college education and above, mostly Caucasians, and mostly married. The mean body mass index was 28.6 indicating overweight on average, and 56% were postmenopausal. Most participants had Stage I or II breast cancer with an estrogen and progesterone receptor positive tumor. 

### 3.2. Descriptive Levels of Biobehavioral Factors

Table 2 includes the range of the scores, mean values with standard deviations on major variables of the study. Cancer-specific stress showed a wide range of the scores, 0–65, with the moderate level of stress, on average. Similarly, mood disturbance scores varied widely among the participants with a moderate mean score. Symptom scores seemed relatively low at baseline, time of which was prior to the beginning of cancer adjuvant therapy. The quality of life score was relatively high at this baseline. Total leukocyte counts were high on some participants, but the mean value stayed within normal range. Similar patterns were shown for neutrophil and lymphocyte percentages to indicate a wide range but the within-normal mean values. The average NLR remained moderate, but the score range indicated a high NLR in some participants.

### 3.3. Correlations between Biobehavioral Factors

 As can be seen in Table 3, cancer-specific stress showed relatively high significant positive associations with mood disturbance and symptom perception but a significant negative association with quality of life. For associations with the immune responses, cancer-specific stress or symptom perception did not show any significant associations. In contrast, mood disturbance showed significant positive associations with total leukocyte count, neutrophil %, and NLR but a significant negative association with lymphocyte %, whereas QOL showed the opposite associations.

## 4. Discussion

The primary purpose of this study was to assess the levels of cancer-specific stress and related mood disturbance in women shortly after diagnosis of breast cancer and what associations these factors had with symptom perception, quality of life, and biological responses of immune cell counts. 

### 4.1. Stress, Mood Disturbance, and Negative Clinical Outcomes

Participants, on average, showed the moderate levels of cancer-specific stress, which were highly correlated with increased mood disturbance. Although the average level of mood disturbance was only moderate, the range of the scores indicated that some women were reporting a significantly high level of mood disturbance as it was the case for cancer-specific stress. These findings are in line with the findings of previous studies reporting cancer diagnosis being a major source of psychological and emotional stress [1–4]. Although initial psychological and emotional stress following cancer diagnosis tends to decline over time, a significant proportion of patients also continue to experience high levels of stress even years after the successful completion of cancer treatment [1, 5]. Some patients may further develop posttraumatic stress disorder precipitated by cancer diagnosis [6, 7], indicating the various levels of different individualized responses to a similar stressor of cancer diagnosis. These findings suggest the importance of assessing stress from the individual patient perspective. A significant additional concern is that nearly half of these stressed patients had neither sought nor intended to seek any professional psychosocial support [51], raising the possibility of prolonging stress, which can compromise their long-term health outcomes.

Mood disturbance, including depression and anxiety, has been highly correlated with psychological stress and, as a result, mood disturbance has been used frequently to indicate psychological stress [8, 9]. Consistent with previous findings, a correlation between cancer-specific stress and mood disturbance was high in this study. Both measures, in turn, were positively correlated with patients' perception of worse symptoms and inversely correlated with quality of life. These relationships seem to be somewhat stronger with mood disturbance than cancer-specific stress. A possible explanation is that stress assessment in this study was narrowly limited to cancer-specific stress only, whereas mood disturbance may represent the feeling more broadly to include potential mood disturbance from noncancer events occurring simultaneously. 

The significant positive relationship between depression and symptoms was reported previously by other studies with various cancer populations, including women with breast cancer [10, 12, 14] and patients with advanced cancer [11]. Causal relationships were demonstrated in that pretreatment emotional distress significantly predicted postchemotherapy fatigue [52]. In addition, high psychological distress was associated with low physical and psychological quality of life in women with breast cancer [3, 23–25]. Initial distress was found to be the most potent predictor for long-term quality of life in a breast cancer population [24]. Furthermore, in animal models of cancer, stress was found to decrease the efficacy of or resistance to chemotherapeutic agents [27, 28]. These findings collectively suggest the importance of early screening of psychological and emotional stress in cancer patients shortly after cancer diagnosis and intervene with the appropriate consultation and referral. Otherwise, high stress may lead to more long-term negative consequences beyond the negative perception of worse symptoms and poor quality of life. 

Several long-term follow-up studies have documented such extended negative impacts of psychological and emotional stress. In women with advanced disease of metastatic or recurrent breast cancer, women who had experienced one or more major stressful or traumatic life events had a significantly shorter disease-free interval than women without such stressful life event. The group difference of median disease-free interval between 31 months and 62 months seems highly significant clinically [26]. Although not all studies showed the same levels of associations, depression in general has been associated with higher cancer recurrence and cancer mortality [17–19]. Furthermore, the causal relationship is indicated in that high early psychological and emotional stress assessed after primary surgery predicted a shorter recurrence-free and overall survival over a median follow-up period of 12.9 years in a breast cancer population [16]. Taken together, a collective body of literature seems to suggest multiple negative associations with and/or consequences from high early psychological and emotional stress in cancer trajectory.

### 4.2. Stress, Immune Responses (Leukocyte Count), and Cancer

 Biological mechanisms underlying the relationship of psychological and emotional stress and negative health outcomes have been assessed in the studies of psychoneuroimmunology. Several reviews and individuals studies indicate that psychological stress induces significant alterations in various immune cell responses in healthy and sick populations [25, 29–32, 53]. Stress reduction in cancer trajectory or from behavioral interventions was associated with improved immune responses in women with breast cancer [33, 54]. These findings suggest that behavioral interventions of stress management may improve biological response profiles, potentially protecting the patients from long-term negative health consequences. 

Because immune cell functions are likely to be influenced by altered immune cell counts, numeric assessments of immune cell counts may provide the early useful information. Acute and chronic stress can induce leukocyte redistribution [37], but most investigations of stress and immune cell counts were focused on lymphocytes and T cell subset changes [55, 56]. For neutrophil and lymphocyte redistribution, stress hormones play a role. Glucocorticoid hormones (e.g., cortisol) induce a significant reduction in lymphocyte counts in circulation, whereas catecholamines seem to induce an increase in neutrophil counts but a decrease in lymphocyte counts [37–39]. 

 Despite that neutrophils are the largest leukocyte constituent in circulation and directly involved in host defense and inflammation [34], neutrophils have been rarely examined in relation to stress. Academic stress was associated with a significant increase in the release of neutrophil superoxide, a proinfiammatory substance [35, 36], suggesting a potential contribution of neutrophil function to inflammation under stress. When acute short-term stress challenge (16–20 minutes) was applied in a controlled laboratory setting, the lymphocyte percentage increased with a concomitant decrease in neutrophil percentage during stress challenge. Immediately following stress challenge, however, lymphocyte percentage continuously declined while neutrophil percentage climbed beyond the baseline during an hour poststress period in college students [57]. 

It is particularly interesting to note that several investigators have documented a clinical significance of the NLR in long-term cancer outcomes. Elevated preoperative NLR, particularly a value greater than 4 or 5, was predictive of overall and cancer-specific survival and cancer recurrence in colorectal cancer patients [40, 41] and patients with malignant mesothelioma [42]. Also in other cancers, an elevated NLR was found to be an independent predictor for survival in a large sample of patients with various types of cancer [43].

## 5. Conclusions

In summary, the findings of this study indicate that cancer diagnosis triggers a wide range of cancer-specific stress and mood disturbance in women shortly after diagnosis of breast cancer. Psychological and emotional stress, in turn, has negative implications for increased symptom perception and poor quality of life, further adding to the patient suffering. In addition, psychological and emotional stress is significantly associated with increased neutrophil percentage but decreased lymphocyte percentage leading to the increased NLR, which has been associated with negative cancer outcomes in the literature.

## 6. Implications for Practice

The potential impact of psychological and emotional stress is multifold, including unfavorable biobehavioral responses. Screening for cancer-related stress and emotional mood disturbance is essential and must be part of routine assessment in cancer nursing practice. Subsequently, patients in high distress should be guided to proper resources, such as a variety of stress management and behavioral interventions. At the same time, it is important for nurse scientists and practitioners to test and compare the efficacy of various behavioral interventions using a biobehavioral approach, the findings of which will refine the evidence-based practice in stress management. 

## Figures and Tables

**Table 1:** Participant characteristics (*N*=100).

Variable	Mean (SD)	*n* (%)
Age (years)	48.8 (8.5)	
Education		
High school and other		30 (30.0)
Bachelor's degree/some college		51 (51.0)
Some graduate school/graduate degree		19 (19.0)
Ethnicity		
African American		23 (23.0)
Native American		2 (2.0)
Caucasian		75 (75.0)
Marital status		
Single		6 (6.0)
Married or living as married		73 (73.0)
Separated/divorced/widowed		19 (19.0)
Missing		1 (1.0)
Menopausal status		
Premenopause		30 (30.0)
Postmenopause		56 (56.0)
Perimenopause		14 (14.0)
Missing		10 (10.0)
Body mass index	28.6 (6.2)	
19–24.9		33 (33.0)
25–29.9		24 (24.0)
30–46.6		33 (33.0)
Missing		10 (10.0)
Stage (TNM classification)		
I		24 (24.0)
IIA		42 (42.0)
IIB		14 (14.0)
IIIA-IV		17 (17.0)
Missing		3 (3.0)
Estrogen/progesterone receptor		
+/+		69 (69.0)
−/−		22 (22.0)
Mixed		2 (2.0)
Missing		7 (7.0)

**Table 2:** Descriptive values of study variables.

	Possible range	Score range	Mean	SD
Cancer-specific stress	0–75	0–65	30.5	14.3
Mood disturbance	0–148	4–122	44.5	22.7
Symptoms	0–4	0–1.43	.57	.32
Quality of life	0–160	73–148	115.8	17.0
WBC (×1000/*μ*L)		3.2–11.7	6.7	1.7
Neutrophil %	0–100	39.0–77.0	59.9	9.3
Lymphocyte %	0–100	14.0–49.0	28.8	8.1
NLR		.8–5.0	2.4	1.0

WBC: total leukocytes; NLR: neutrophil-to-lymphocyte ratio; SD: standard deviation.

**Table 3:** Correlations between study variables.

	Cancer stress	Mood disturbance	Symptoms	QOL
Mood disturbance	.57**	—		
Symptoms	.45**	.54**	—	
QOL	−.43**	−.56**	−.45**	—
WBC	−.08	.25*	−.00	.02
Neutrophil %	.08	.39**	.14	−.37**
Lymphocyte %	−.08	−.34**	−.18	.43**
NLR	.04	.40**	.18	−.39**

**P ≤ .01, *P ≤ .05. QOL: quality of life; WBC: total leukocytes; NLR: neutrophil-to-lymphocyte ratio.
